# Replies to the commentaries on the question of ‘Is it time to abandon the biological species concept?’

**DOI:** 10.1093/nsr/nwaa117

**Published:** 2020-06-03

**Authors:** Chung-I Wu, Xinfeng Wang, Ziwen He, Suhua Shi

**Affiliations:** State Key Laboratory of Biocontrol, Guangdong Key Lab of Plant Resources, School of Life Sciences, Sun Yat-Sen University, China; State Key Laboratory of Biocontrol, Guangdong Key Lab of Plant Resources, School of Life Sciences, Sun Yat-Sen University, China; State Key Laboratory of Biocontrol, Guangdong Key Lab of Plant Resources, School of Life Sciences, Sun Yat-Sen University, China; State Key Laboratory of Biocontrol, Guangdong Key Lab of Plant Resources, School of Life Sciences, Sun Yat-Sen University, China

In reassessing the biological species concept (BSC), the central disagreement is whether the concept of species should be linked to the process of species formation. Here, we argue that species should be, and can only be, defined in the context of the process of speciation. By evaluating BSC this way, we find the large body of genomic evidence on ‘speciation with gene flow’ does not constitute sufficient evidence to reject BSC. Whether and how BSC should be continually re-examined in light of the genomic evidence will be the crux of the debate on species and their origin.

## WHAT IS, AND IS NOT, IN THE DEBATE

We first clarify what the debate is and is not, as initiated by Wang *et al*. [1]. The debate is only about the biological species concept (BSC) [2,3], and is not about any of the many other species concepts. By using a provocative question in the title, Wang *et al*. may have led to the suspicion of advocating for an alternative concept [4–6] but that was not the focus. (While Wang *et al*. did use an alternative ‘genic view’ model by Wu [7], it only serves as an alternative hypothesis in a statistical test, aiming to inform about the procedures of testing the null hypothesis, i.e. BSC.)

Given the centrality of BSC in evolutionary biology, we should rigorously and regularly re-examine this concept to make sure it should still remain in the center court. The efforts are to be focused on falsifying BSC as the null model. Our answer to the question in the title is ‘Not yet’, the same as the one reached in ref. [4]. The difference is that we believe BSC is falsifiable but Butlin and Stankowski do not.

## THE CENTRAL DISAGREEMENT—PROCESS VERSUS OUTCOME, OR SPECIATION VERSUS SPECIES

Wang *et al*. [1] reviewed the published genomic evidence that could be used to reject BSC. In doing so, we assumed that there is a way to disprove BSC if one can link the process of species formation with the outcome of speciation (i.e. the delineation of species). For BSC, this process is allopatric speciation, which requires the absence of gene flow during speciation. The mechanism that prevents gene flow is usually geographical isolation.

This linking of process and outcome in testing BSC is the central disagreement in this debate. The two commentaries [4,6] both suggest that species are the products of a process and it is incorrect to link the process to the definition of a product. It would be true if the making of species can be compared to the making of cars. One does not have to know how Italian sports cars are made in order to tell them apart from American SUVs. But this product versus process dichotomy is a false argument. A car is clearly defined as the machine that rolls off the assembly line to be driven away. Before that, the machine is not a car. In the ‘making’ of species, the products are always there, fully assembled at all times.

An appropriate analogy to species and speciation is the development of a child into an adult. Delineating the two stages, much like delineating two taxa as two species, has to incorporate the transitional period into consideration. A person may develop the various adult characteristics unevenly at different ages and, hence, a teenager is somewhere between a child and an adult. The delineation of adulthood versus childhood hence requires an understanding of the process of human development.

The formation of species also progresses through the gradual transitions in various reproductive, behavioral, morphological and physiological characters, all of which are quantitative in nature. The statement that ‘two taxa are reproductively isolated’ is rather imprecise since RI (reproductive isolation) is not an on–off switch. If the F1 males between two taxa are 100% sterile and F1 females are 95% fertile, are the species reproductively isolated? This sort of half-way RI is the rule, rather than the exception, in most cases where the species status is not obvious [3,8]. The gradation is true for morphology, behavior and physiology as well [3].

These concerns force us to ask a deeper question: why do we need to define species? For a taxonomist or a conservation biologist with a need to classify animals and plants, BSC has played little role in the actual classification of species. Instead, it is suggested that BSC sets up the framework, within which we can attempt to understand the process of evolution and, in particular, speciation. Hence, BSC is the guiding principle [4,6].

We would like to propose a radical departure from the approach of ‘developing a species concept in order to study the process of species formation’. It would seem more logical that we try to understand the process of speciation and, with that understanding, decide on where to draw a line (in fact, a fat band) in the process to mark the completion of speciation. Similarly, the adulthood–childhood dichotomy is built on the understanding of the developmental process as well. A demarcation is then imposed on the process to separate the adulthood and childhood. The delineation timing would depend on why we need to define adulthood (for marriage, for voting, for alcohol consumption, etc.).

Mayr [2] has emphasized that species, unlike genus or higher taxonomic ranks, is a biological reality. We may take this view a step forward – the biological reality is the process of speciation and the concept of species is built on that reality. Regardless of how one views the relationship, the species concept and the process of speciation are inseparable.

## Can species delineation be separated from the process of speciation?

BSC can be delinked from the process of speciation only if the transition period (from population differentiation to species separation) is very brief. There is no extended process, which is compressed into a time point. This possibility is, however, remote as the characters pertaining to species distinction (reproductive, behavioral, morphological, etc.) are continuously graded over a long time-span. For example, between full reproductive compatibility and complete reproductive isolation lies a long series of partial reproductive incompatibilities. Even the three sibling species of *Drosophila melanogaster* that are unambiguously distinct species exhibit partial RI whereby F1 females and backcross F2 females are nearly fully fertile. In contrast, hybrid males are strongly sterile in any form of hybridizations [9–12]. Full RI evolved much later between *D. melanogaster* and the trio. The transitional period is longer than the age of many species. Again, BSC is a species concept that is critically dependent on the process of speciation.

## THE DISAGREEMENT OVER LINKING BSC TO ALLOPATRIC SPECIATION

Does BSC then have to be linked to the allopatric mode of speciation? The linking is obvious in the literature since Mayr's days [2,3,7,13]. Given the strong view on genetic cohesion, BSC would have to rely on geographical isolation to suppress gene flow before genetic changes can lead to RI [1,3,7,14,15]. Furthermore, the evolution of RI, especially post-mating RI, is much more likely in the absence of gene flow, whereas the parapatric and sympatric modes both permit gene flow [3,15–21]. It is thus unsurprising that Mayr (1963 and later writing) opposed sympatric speciation and downplayed the importance of parapatric speciation. The latter, as cogently argued by Endler [22], might be the prevalent mode of speciation.

If BSC is decoupled from the allopatric mode of speciation, we may ask ‘what would BSC become?’ It would be a concept whereby species are separate gene pools that are reproductively isolated from one another [3,13,23]. So defined, BSC would skip the transitional period by defining species only after all aspects of speciation are completed. At that late stage, there is no hybridization and no gene flow, even in sympatry.

Nevertheless, a species concept should help resolve the difficult issues of species delineation, rather than take up only the obvious cases and leave the rest unexplained. By the stringent requirement of complete RI, the three extensively examined and well-defined sibling species of *Drosophila* (*D. simulans*, *D. mauritiana* and *D. sechellia*) should be considered one single species. If so interpreted, BSC would be close to the concept of a syngameon [24–26], a cluster of loosely connected gene pools that are not connected to any others. Decoupling BSC from allopatric speciation therefore would lead to all sorts of inconsistencies.

## THE DISAGREEMENT OVER CASES OF ‘SPECIATION WITH GENE FLOW’ IN RELATION TO BSC

We suggest that BSC should be accepted or rejected together with allopatric speciation. Wang *et al*. [1] surveyed a large literature that shows speciation having occurred under gene flow. While this literature may be used against BSC, we point out that BSC is not inconsistent with speciation with gene flow, especially if the gene flow happens at the start of the speciation process. The evidence against BSC should be the continual gene flow until speciation is complete. We find no convincing evidence in this regard as studies generally show gene flow near the beginning of speciation.

We recognize that we could not infer the timing of gene flow in every study. Such inferences demand a thorough understanding of the data, not privy to non-authors. Gao and Rieseberg indicated that their studies [6,27,28] have offered sufficient evidence to reject the BSC that we looked for. If more studies, upon careful evaluation, will offer convincing evidence against BSC, then the verdict against BSC should be ready.

Nevertheless, the proof of late-stage gene flow during speciation is much more demanding than simply finding some evidence of gene flow. Wang *et al*. [1] offer a model for the expected genomic patterns if gene flow continues toward the end of speciation. Clearly, we need more elaborate models against which the ‘expanded’ BSC can be tested.

## CONCLUSION

In Mallet's commentary, an alternative species concept is proposed but the current debate focuses on BSC [5]. When we do need alternative concepts, as Mallet anticipates [5], we suggest that they be linked to a process of speciation. The process is the biological reality while species is, in essence, a concept. Finally, we quote Butlin and Stankowski ‘that the BSC is highlighted in every biology textbook and lecture course, more than 80 years after it was introduced and formalized’ [4]. While this could be the raison d’être for accepting BSC, we would argue that, precisely because of its longevity and popularity, BSC needs to be regularly and rigorously tested. After all, doubts have been constantly expressed [7,29–33] while the textbooks continue to put BSC at the center of evolutionary biology [13,34].

## References

[1] Wang X , He Z, Shi S et al. Natl Sci Rev 2020; 7: 1387–97. 10.1093/nsr/nwz220 PMC8288927 34692166

[2] Mayr E . Animal Species and Evolution. Cambridge: Harvard University Press, 1963.

[3] Coyne JA , Orr HA. Speciation. Sunderland: Sinauer Associates, Inc., 2004.

[4] Butlin RK , Stankowski S. Natl Sci Rev 2020; 7: 1387–97. 10.1093/nsr/nwaa109 PMC8288981 34692168

[5] Mallet J . Natl Sci Rev 2020; 7: 1401–7. 10.1093/nsr/nwaa116 PMC8288880 34692169

[6] Gao L , Rieseberg LH. Natl Sci Rev 2020; 7: 1398–400. 10.1093/nsr/nwaa108 PMC8288963 34692167

[7] Wu C-I . J Evol Biol 2001; 14: 851–65.

[8] Wu C , Davis AW. Am Nat 1993; 142: 187–212. 19425975 10.1086/285534

[9] Ting CT , Tsaur SC, Wu ML et al. Science 1998; 282: 1501–4. 9822383 10.1126/science.282.5393.1501

[10] Wu C , Palopoli MF. Annu Rev Genet 2003; 28: 283–308. 10.1146/annurev.ge.28.120194.001435 7893128

[11] Sun S , Ting CT, Wu C-I. Science 2004; 305: 81–3. 15232104 10.1126/science.1093904

[12] Sawamura K , Davis AW, Wu C-I. Proc Natl Acad Sci USA 2002; 97: 2652–5. 10.1073/pnas.050558597 PMC15984 10706624

[13] Futuyma DJ , Kirkpatrick M. Evolution. Sunderland: Sinauer Associates, Inc., 2017.

[14] Yang M , He Z, Shi S et al. Mol Ecol 2017; 26: 2845–9. 28345182 10.1111/mec.14117

[15] He Z , Li X, Yang M et al. Natl Sci Rev 2019; 6: 275–88. 31258952 10.1093/nsr/nwy078 PMC6599600

[16] Mallet J . Trends Ecol Evol 2005; 20: 229–37. 16701374 10.1016/j.tree.2005.02.010

[17] Barton NH , Hewitt GM. Annu Rev Ecol Syst 1985; 16: 113–48.

[18] Strasburg JL , Rieseberg LH. Evolution 2008; 62: 1936–50. 18462213 10.1111/j.1558-5646.2008.00415.x PMC2601659

[19] Payseur BA , Rieseberg LH. Mol Ecol 2016; 25: 2337–60. 26836441 10.1111/mec.13557 PMC4915564

[20] Seehausen O . Trends Ecol Evol 2004; 19: 198–207. 16701254 10.1016/j.tree.2004.01.003

[21] Abbott R , Albach D, Ansell S et al. J Evol Biol 2013; 26: 229–46. 23323997 10.1111/j.1420-9101.2012.02599.x

[22] Endler JA . Geographic Variation, Speciation, and Clines. Princeton: Princeton University Press, 1977. 409931

[23] Butlin RK , Galindo J, Grahame JW. Philos Trans R Soc B 2008; 363: 2997–3007. 10.1098/rstb.2008.0076 PMC2607313 18522915

[24] Lotsy JP . Evolution by Means of Hybridization. The Hague: M. Nijhoff, 1916.

[25] Grant V . Plant Speciation. New York: Columbia University Press, 1971.

[26] Suarez-Gonzalez A , Lexer C, Cronk QCB. Biol Lett 2018; 14: 20170688. 29540564 10.1098/rsbl.2017.0688 PMC5897607

[27] Sambatti JBM , Strasburg JL, Ortiz-Barrientos D et al. Evolution 2012; 66: 1459–73. 22519784 10.1111/j.1558-5646.2011.01537.x

[28] Yatabe Y , Kane NC, Scotti-Saintagne C et al. Genetics 2007; 175: 1883–93. 17277373 10.1534/genetics.106.064469 PMC1855124

[29] Donoghue MJ . Bryologist 1985; 88: 172–81.

[30] Paterson HEH . The recognition concept of species. In: Species and Speciation. Pretoria: Transvaal Museum, 1985, 21–9.

[31] Templeton A . Otte D and Endler JA. Speciation and Its Consequences. Sunderland: Sinauer Associates, Inc, 1989, 3–27.

[32] Mallet J . Trends Ecol Evol 1995; 10: 294–9. 21237047 10.1016/0169-5347(95)90031-4

[33] Wu C , Ting CT. Nat Rev Genet 2004; 5: 114–22. 14735122 10.1038/nrg1269

[34] Ridley M . Evolution. Malden: Blackwell Science, 2004.

